# Management of high-energy tibial pilon fractures

**DOI:** 10.1007/s11751-015-0231-5

**Published:** 2015-09-25

**Authors:** Nebu Jacob, Amit Amin, Nikolaos Giotakis, Badri Narayan, Selvadurai Nayagam, Alex J. Trompeter

**Affiliations:** Department of Trauma and Orthopaedic Surgery, St Georges Healthcare NHS Trust, Blackshaw Road, Tooting, London, SW17 0QT UK; Limb Reconstruction Unit, Department of Trauma and Orthopaedic Surgery, Royal Liverpool and Broadgreen University Hospital NHS Trust, Liverpool, L7 8XP UK; 1 Locke Gardens, Slough, Berkshire SL3 7BE UK

**Keywords:** Pilon fractures, Management, Strategy, Algorithm, Reconstruction, Bone defects

## Abstract

Tibial pilon fractures result from high-energy trauma unlike usual ankle fractures. Their management provides numerous challenges to the orthopaedic surgeon including obtaining anatomic reduction of articular surface and the management of associated soft tissue injuries. This article aims to review major advances and principles that guide our practice today. We also discuss a treatment algorithm based on a staged approach to the fracture: initial spanning external fixation followed by definitive fixation.

## Introduction

Pilon is the French word for a pestle. Etienne Destot, a French Radiologist, is credited for using the term to describe the fracture in 1911. He compared the talus to a pestle. High-energy tibial ‘pilon’ fractures are due to axial loading with the talus driven into the distal tibia, exploding the distal tibial articular surface with impaction of the comminuted metaphyseal bone, and with occasional proximal diaphyseal extensions. These commonly result from falls from a height or from motor-vehicle-related accidents [1]. The degree of trauma to the surrounding soft tissue envelope cannot be underestimated; there is limited muscle cover between the skin and bone at this level of the lower limb, and the energy of the injury is transferred directly to these soft tissue structures. Open fractures are common, and even in the absence of an open lesion, significant soft tissue damage must be appreciated in closed injuries [2].

The treatment objectives are to restore articular congruency and mechanical alignment and to allow early functional rehabilitation whilst minimising soft tissue complications. Two-stage management with initial spanning external fixation allows soft tissue resuscitation prior to definitive management and has gained acceptance by most surgeons [1, 3–7].

Microscopic articular cartilage damage that occurs at the time of injury has significant bearing on the long-term prognosis even in the presence of anatomical joint reduction [8, 9]. The challenge lies in minimising complications, such as deep infection, whilst optimising clinical outcome through appropriate and well-timed surgery. This article focuses on the controversies in the management of high-energy pilon fractures, and we present a treatment algorithm based on the authors’ collective clinical experience.

## Classification

The two main X-ray classification schemes are those of Ruedi and Allgower [10] and the AO Foundation and Orthopaedic Trauma Association (AO/OTA) [11].

**Fig. 1:**
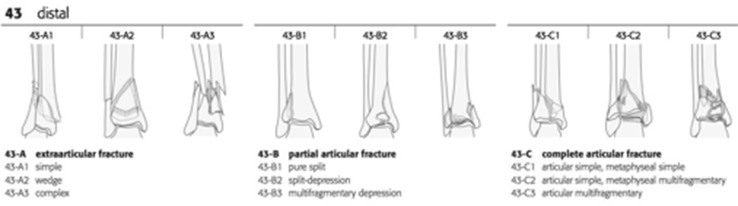
AO classification of distal tibial fractures (Müller AO Classification of Fractures-Long Bones, Copyright by AO Foundation, Switzerland)

Ruedi and Allgower described three groups, specific to tibial pilon fractures, based on the size and displacement of articular fragments: type I represents non-displaced intra-articular fractures without loss of articular congruency; type II represents displaced fractures with loss of articular congruency; and type III represents those severely comminuted fractures with impaction of the distal tibia.

**Fig. 2:**
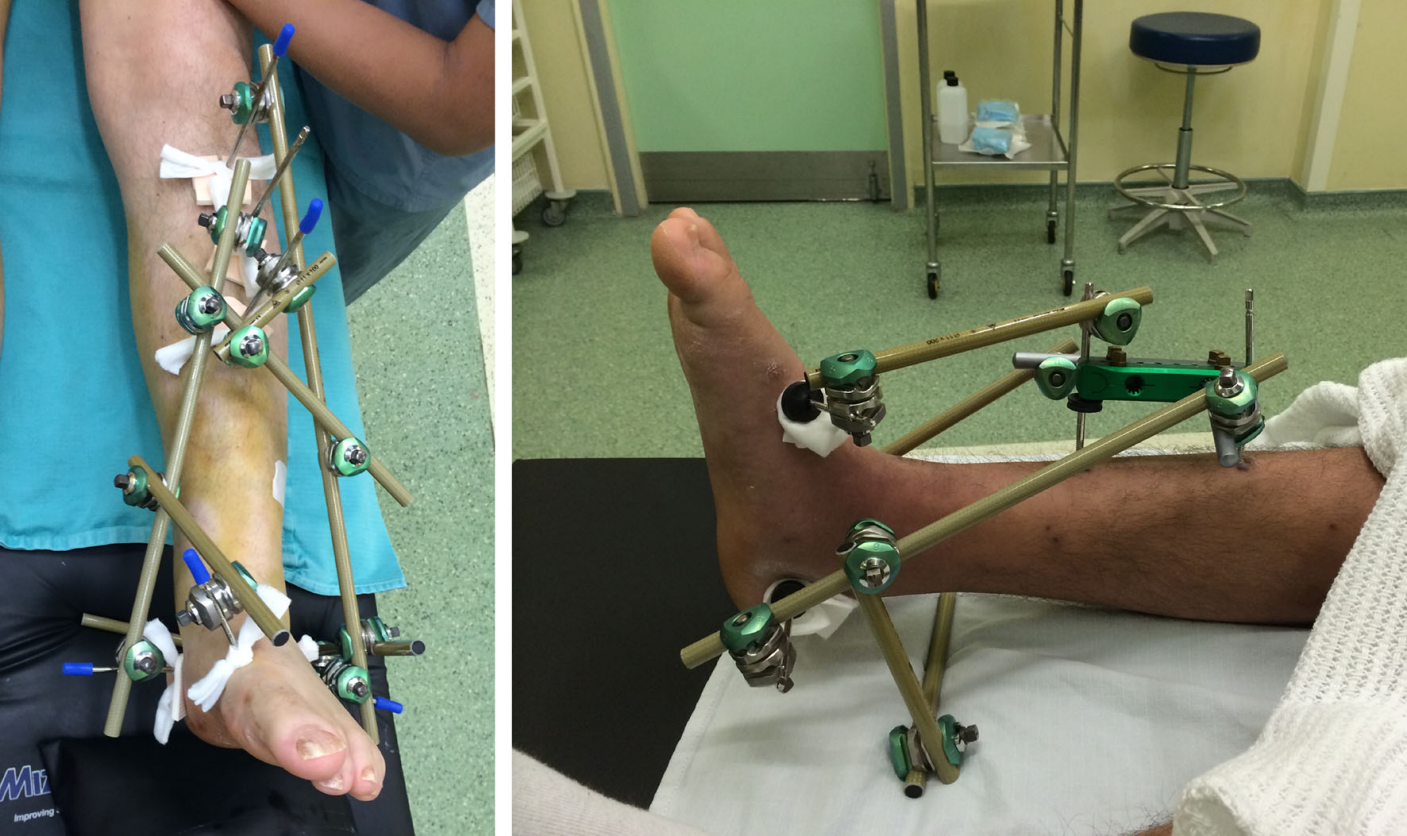
Temporary external fixator configuration for damage control

The AO/OTA group use an alphanumeric system to describe all fractures. The first number represents the bone: in this case, tibia is ‘4’; the second represents the segment of bone which in this case is ‘3’ for distal. Following this, ‘A’ represents extra-articular fractures within 5 cm of the ankle joint and ‘B’ represents partial articular injuries, both not included in this review. Group ‘C’ denotes complete articular injury where there is no direct continuity between the diaphysis and the articular segment. This group accounts for the majority of high-energy pilon fractures. The final numbers in this classification represent the subgroups of each type and refer to the degree of comminution of the articular component and the metaphysis (Fig. 1).

**Fig. 3:**
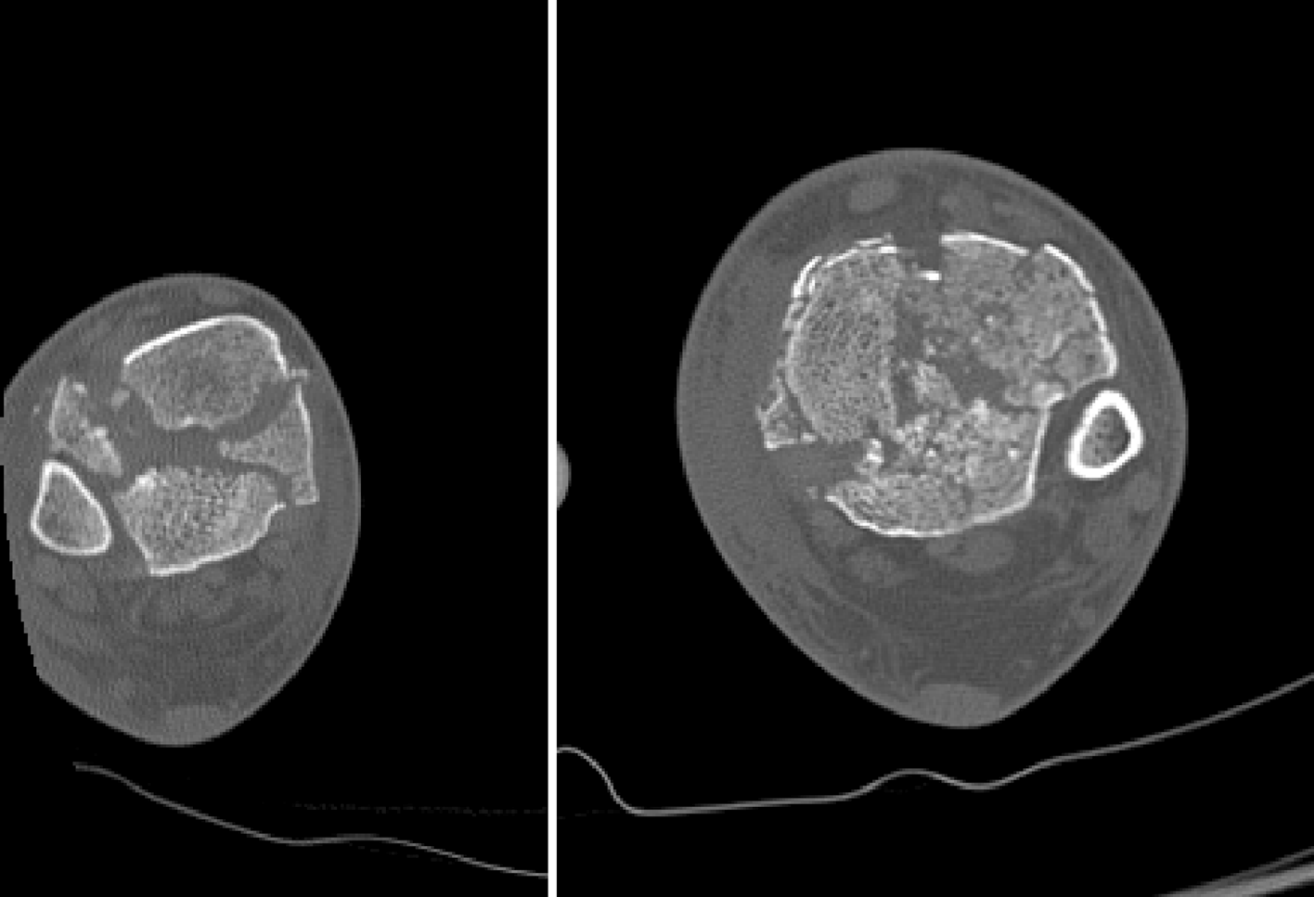
Articular fragments with varying degrees of comminution as seen on axial CT views

Swiontkowski et al. [12] raised concern about classification systems in general when reporting on the inter-observer reliability of the AO/OTA system. They found moderate correlation for groups A, B or C and poor correlation between subgroup detection. They concluded that compartmentalising fracture severity, which behaves as a continuous and not a dichotomous variable, should be avoided.

Topliss et al. [13] reviewed a consecutive series of 126 pilon fractures with 115 cases classified as AO/OTA ‘C’ type injuries. Of these, 67 patients (52 %) had the more complex C3 injuries. It is this subgroup, which comprises true high-energy pilon fractures, where significant discrepancy and disagreement exist in the literature over management. Their study provided a CT-based classification segregating fracture patterns into two main families, which were termed ‘sagittal’ and ‘coronal’ based on the primary fracture line seen on axial cuts at the level of the plafond. These subtypes were assessed for patient and deformity characteristics, noting that sagittal plane fractures tended to present in varus and had resulted from higher-energy injuries in younger individuals. The coronal plane fractures tended to present in valgus and were associated with lower-energy injuries in older patients. This study offered an interesting insight into the spectrum of fracture pattern variability. Although the authors reported good inter-observer reliability, their findings have yet to be replicated.

**Fig. 4:**
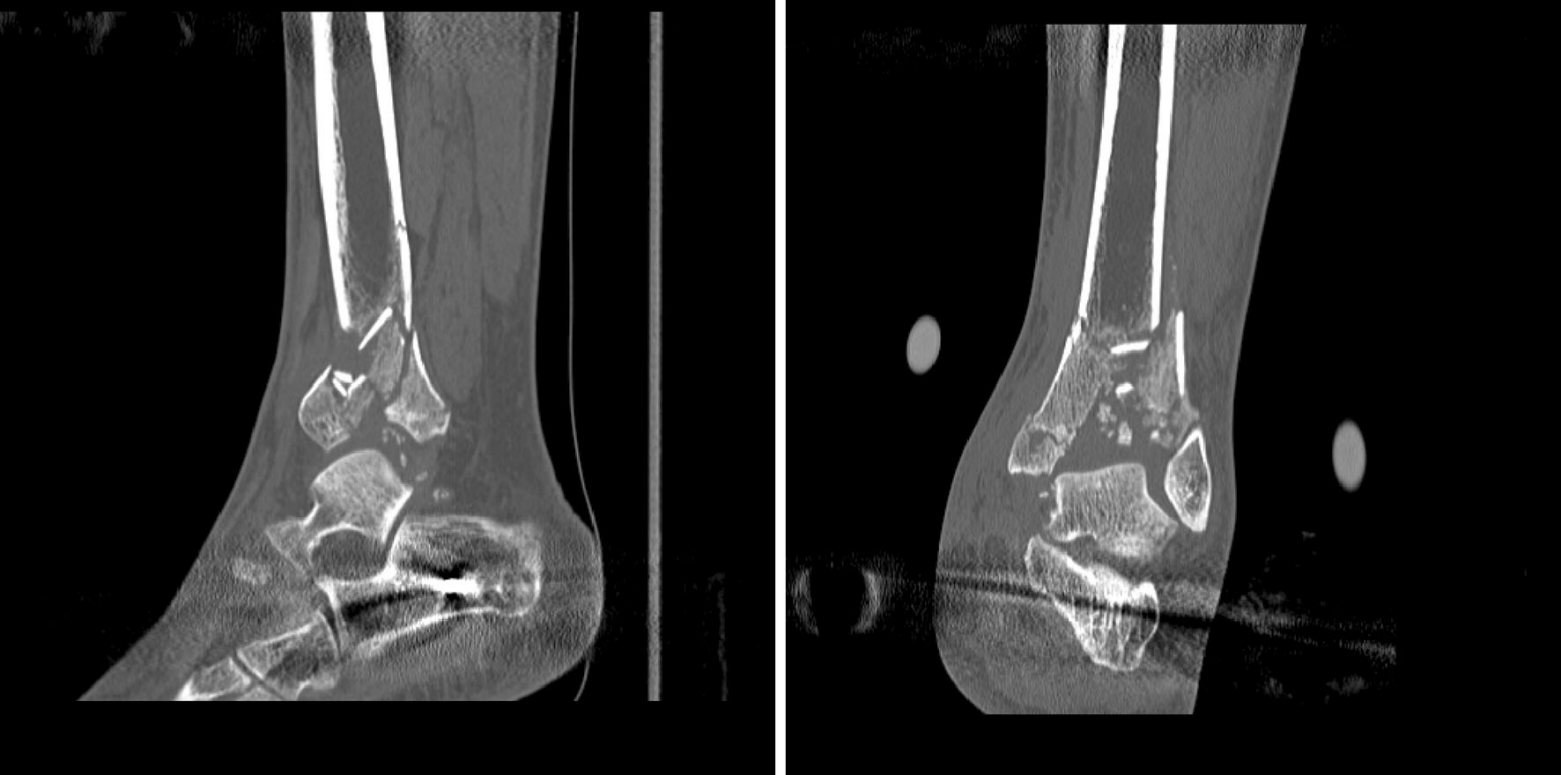
Anterior and central areas are most often comminuted, and central die-punched fragments can be appreciated on both axial and reformatted sequences on CT

## Initial management

### Pilon fractures managed akin to polytrauma with damage control strategies

Early operative management through a tenuous soft tissue envelope risks wound healing problems, invites infection and can potentially lead to limb amputation. Temporary spanning external fixation, with or without fibular stabilisation at index surgery, has gained acceptance as the first-line intervention and is considered a local ‘damage control’ strategy (Fig. 2).

Patterson and Cole [5] first described the two-stage management of pilon injuries with definitive management undertaken at 10–14 days following external fixation and with all patients having formal open reduction and internal fixation. Sirkin et al. [3] popularised this protocol in two subsequent publications stating that the technique was successful in both closed and open fractures.

**Fig. 5:**
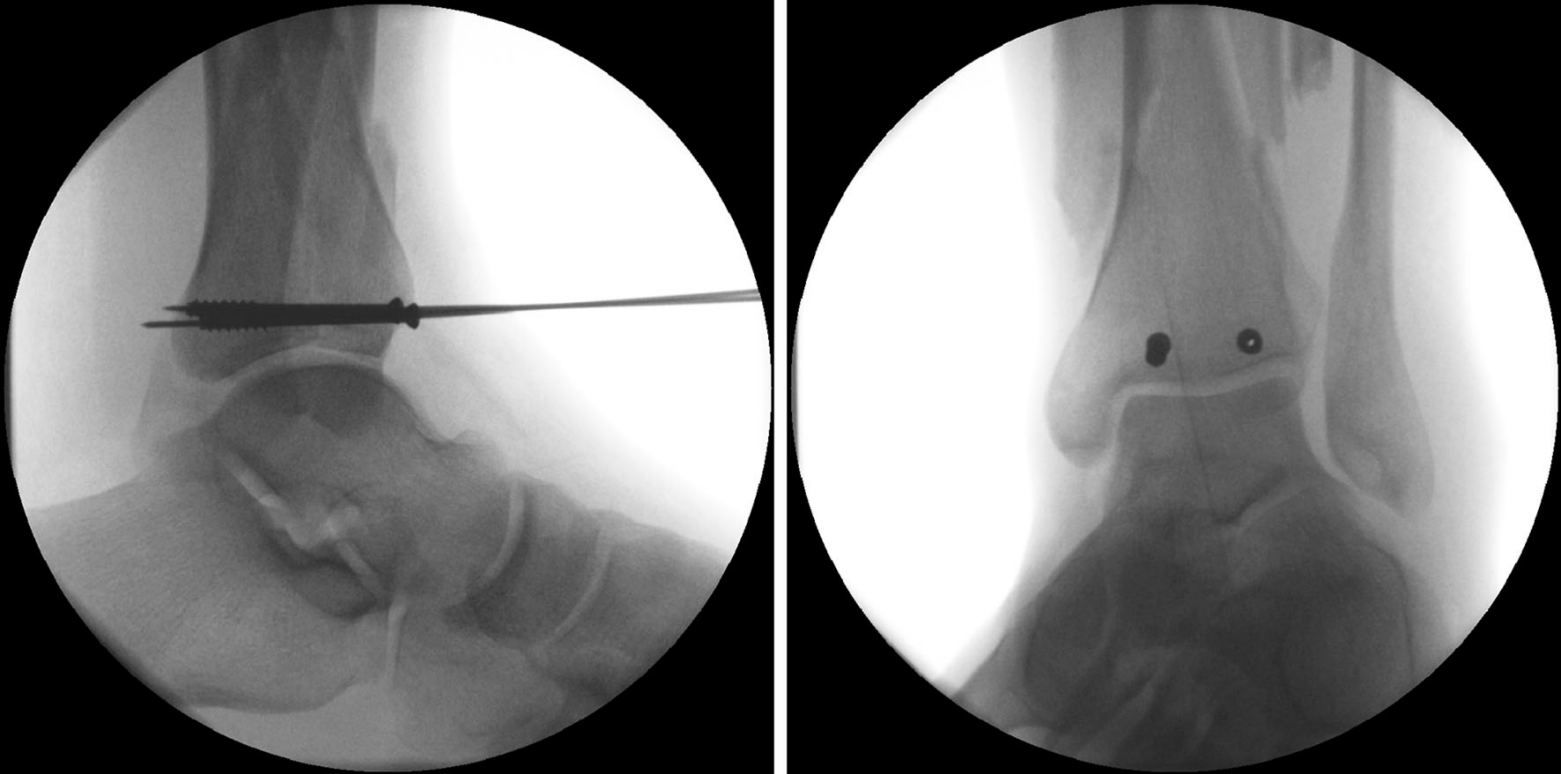
Cannulated partially threaded screws used to reconstruct the articular surface

Temporary stabilisation should be performed as soon as possible but preferably during daylight hours on a designated operating list. A careful restoration of alignment with the external fixator must be considered at this early stage. Fixator constructs vary with ‘delta’ and ‘A’ frames assemblies being most common. An extension of the fixator onto the forefoot (usually the first metatarsal) is helpful to avoid an equinus contracture. This method of skeletal stabilisation has superseded calcaneal traction as it permits patient mobilisation albeit non-weight bearing. In some centres, patients are sent home whilst awaiting soft tissue recovery and definitive management.

### CT scan

CT scanning is a prerequisite for planning definitive management and is best performed after application of spanning external fixation and restoration of overall alignment through ligamentotaxis. The axial cuts at the level of the plafond accurately define fracture plane orientation, whilst sagittal and coronal reformatting allows a full assessment of fracture morphology.

Tornetta et al. [14] correlated radiographs and CT scans in 22 patients with pilon fractures. Based on the CT findings, they altered their surgical approach in 64 % of their patients. In 12 patients, the major fracture line exited laterally, and in 10 patients, it exited medially. The identification of this major fracture line dictated the surgical approach to the fracture. Where the fracture line exited medially, an anteromedial approach based on the tibialis anterior was used. In the others with a lateral exit of the fracture line, a lateral approach between the extensor digitorum communis and peroneus tertius was used. The CT scan provided vital information on metaphyseal comminution which in five patients was fixed percutaneously. Images from the CT are essential as axial cuts demonstrate fracture lines common to all pilon fractures, knowledge of which is vital for pre-operative planning, incision placement and articular reduction.

There are three typical articular fragments: anterolateral, posterolateral and medial, with variations in size and comminution (Fig. 3).

Anterior and central areas are most often comminuted and central die-punched fragments are appreciated both on axial and reformatted sequences (Fig. 4).

## Definitive treatment options

The basic principles of definitive treatment are:

1. Articular reduction and stabilisation.
2. Restoration of alignment by reduction in the reconstructed articular block to the diaphysis.
3. Management of bone loss at primary surgery or as a planned late intervention (C3 injuries).
4. Respect for the soft tissue envelope
5. Early restoration of motion

Treatment choice is based upon the severity of the soft tissue injury, fracture pattern and the treating surgeon’s experience. There is no level I evidence currently for optimal management with both internal and external fixation techniques, alone or in combination, commonly employed.

Traditional open reduction and internal fixation (ORIF) of complex type ‘C’ fractures, with direct exposure of the metadiaphyseal region, extensive surgical dissection and handling of all fracture fragments was associated with an unacceptably high soft tissue complication rate [15].The early good results of ORIF, reported by Reudi and Allgower [10], were based on a different patient and fracture population, many of whom sustained lower-energy distal tibial fractures with extension to the tibial plafond secondary to skiing injuries. Nevertheless, their four classic principles of treatment: plating the fibula to length, articular reconstruction, bone grafting of metaphyseal defects and providing a medial buttress to the tibia, still remain important orthopaedic concepts.

The role of initial fibula fixation is controversial. The proposed benefits include restoration of length, indirect reduction in the tubercle of Chaput (anterolateral) and Volkmann (posterolateral) tibial fragments in the case of distal fibula fractures, and a faster soft tissue recovery. Conversely, ignoring the fibula fracture allows the option of tibial shortening to improve fracture contact at the metaphysis, especially in the type C3 fracture where the metaphysis is comminuted and prone to delayed healing. In pilon fractures, Lee et al. [16] found a lower rate of malunion and ankle arthrosis in 6 years of follow-up when the fibula was fixed by plating compared to pin fixation. Rouhani et al. [17] and Williams et al. [18] found no clinical difference at 6-month and at 2-year follow-ups, respectively, in patients treated with ankle-bridging external fixation, with or without fibula plating. The plating group suffered more wound complications, and the non-plating group had more with angular malunion. However, this may have resulted from the bridging technique used in their study which did not provide any direct metadiaphyseal stability.

If fibular fixation is undertaken, careful pre-operative planning for the approach to the tibial pilon and fibula is needed to avoid a high wound complication rate. Ideally, these fractures should to be referred to experts early.

### Restoration of articular surface

Reconstruction of high-energy type C fractures should be performed when the soft tissue conditions allow safe surgical dissection. Direct exposure of the articular segment through planned limited or formal approaches is advocated. Percutaneous techniques can be used with simple articular patterns, but C3 injuries require direct reduction.

The common approaches are either anterolateral or anteromedial depending on the axial CT images at the level of the plafond as described in the study by Tornetta et al. [14]. The incisions allow direct articular reduction, but not definitive fixation. Articular fixation can be accomplished with either two or three 4.0-mm cannulated screws, 4.0-mm partially threaded cancellous or 3.5-mm fully threaded cortical lag screws. Smaller osteochondral fragments can be fixed using variable pitch countersunk screws, mini-fragment plates/screws, or even flush-cut and buried wires (Fig. 5).

Assal et al. describe a formal extensile approach to allowing better visualisation of the articular surface [19]. A 10-cm incision is made starting just lateral to the tibial crest and continues down to the ankle joint, at which point the incision curves medially with an angle of 100°–110°. The saphenous vein and nerve mark the distal extent of the approach. The periosteum of the tibia is incised medial to tibialis anterior, and the entire anterior compartment with the neurovascular bundle is mobilised laterally. This approach suits internal fixation techniques (plating) for definitive management; plates can be introduced through this incision and passed either submuscularly (anterolateral) or subcutaneously (anteromedial, with proximal screw fixation achieved through stab incisions.

### Restoration of the mechanical axis

The restoration of limb length and alignment are also important determinants in the outcome in tibial pilon fractures and can be achieved with internal fixation or definitive external fixation.

### Definitive internal fixation

The evolution of internal fixation techniques over the last 20 years has challenged the Reudi and Allgower principles of early fibula plating and the need for medial tibial buttressing alone. Fractures that end with valgus failure and those with significant anterior comminution are better supported with anterior or anterolateral plating techniques. The coronal ‘family’ of fractures, as described by Topliss et al. [13], if treated with only medial buttress plates, would hold the primary fracture line suboptimally and lead to failure. Furthermore, the incisions required for anterolateral plating often mean that a standard lateral incision for fibula fixation cannot be utilised.

Sirkin et al. [3], in their landmark paper that popularised the staged approach to management, found that in their closed group of pilon injuries of 29 patients, five developed some form of wound necrosis which did not escalate to deep infection; only one patient developed a late complication which was a chronic draining sinus that resolved with fracture consolidation and metal removal. The open fracture group included 17 patients with two late deep infections: one patient underwent limb reconstruction with an aggressive protocol and one patient had a below knee amputation.

The concept of two-stage management is established with a trend towards minimally invasive plating techniques to reduce further wound healing complications. The concept of biological plating with minimally invasive application of pre-contoured implants is a further evolution in internal fixation which enables epiphyseal and metadiaphyseal contact and alignment without extensive periosteal stripping. However, as with any new technology, achieving consistent results requires multiple refinements often with respect to the implant design and surgical technique. Fracture reduction with indirect techniques is more difficult to master, and the view that the implant will compensate for an inadequate reduction will lead to either a mal- or non-union.

Despite an increasing use of ‘biological plating’ in orthopaedic trauma, there is a paucity of evidence on the outcome when applied to patients with ‘C’ type pilon fractures. Most studies refer to a heterogeneous group of patients that include type A and B injuries. Using a two-stage minimally invasive protocol, Borens et al. [20] reported on 17 patients with good to excellent radiographic results at 17-month follow-up although 41 % had developed moderate arthritis at this time. Five of the patients had low-energy trauma, and 12 fractures were classified as either C2 or C3 injuries. This subgroup of higher-energy injuries did not have any serious wound healing problems. The plate used in this study was a non-locking low-profile implant, termed a ‘scallop’ plate, designed to pass through the soft tissues with minimal trauma. Little evidence supports the use of locking plates over standard plates when used in patients with good bone quality. Pre-contoured low-profile non-locking plates, such as those used by Borens et al. [20], can be applied with limited incisions and placed either subcutaneously or submuscularly. These are less bulky and kinder to the soft tissues especially over the medial subcutaneous border of the tibia.

Blauth et al. [21] compared three methods of treatment in a cohort of 51 patients with 47 type C fractures. Twenty-eight patients were treated with one-stage articular reduction and bridging external fixation. Fifteen patients were treated with primary plate fixation, and eight patients had a two-stage minimally invasive intervention, with application of a medial plate when the soft tissues had recovered. The latter option yielded the best results although two comparative groups used in their study, that of definitive bridging external fixation and primary plate fixation are not now considered as reliable management options.

### Definitive external fixation

With increasing comminution of the metaphysis (C3 injuries), restoration of mechanical alignment and achieving stable fixation becomes increasingly difficult. The metaphyseal component of the injury may lead to a non-union or malunion, and these injuries are prone to wound healing complications and infection. Proponents of internal fixation argue that pilon fractures treated by external fixation often result such complications. This leads to the debate surrounding pilon fractures whether definitive management of C2 and C3 injuries are better treated definitively by external or internal fixation. External fixation constructs described in the literature include simple bridging frames, ankle-articulating devices, and hybrid or circular frames which are used mostly in conjunction with limited internal fixation of the articular surface through percutaneous or small incisions [2, 7, 21–23]. The ability of articulating devices to offer useful range of movement during treatment has been questioned and may be due to the difficulty in reproducing movement about the ankle joint axis [24].

The evidence used against external fixation as a valuable definitive option is based largely upon historical techniques where definitive management consisted of bridging the ankle joint with a fixator, without direct control of the metaphyseal component of the injury. Additionally, the more severe injuries are treated with external fixation and introduced a patient selection bias into these studies. Anglen et al. [25] reported dismal results associated with hybrid external fixation when compared to internal fixation for type C fractures. This retrospective study was based on the more severe injuries, including more C2 and C3 types and open injuries that were chosen for treatment with a hybrid fixator as a one-stage intervention. This study demonstrated that one-stage management of high-energy injuries was not effective.

Pin-site infection has been reported to be a serious complication with prolonged external fixation. Whilst this is a recognised complication of fine wire fixation in general, it can be controlled and managed with an integrated multidisciplinary approach [26]. Deep infection rates vary significantly in the literature and are biased by a higher proportion of open injuries treated definitively with external fixation. Papadokostakis et al. [27] reviewed the merits of spanning versus non-spanning frames and found, in their systematic review, that the overall deep infection rate with non-spanning frames was 2.7 %. The deep infection rate in the spanning group was 3.9 % which may be related to the larger proportion of open injuries in this group. The conclusion from this review suggested that there were no statistically significant differences with either technique with respect to infection, non-union or time to union. There was a higher rate of malunion in the spanning group. The groups were heterogeneous, and the relative merits of external fixation as definitive management for these injuries were not determined clearly.

A few studies have reported the outcome of circular ring fixation as definitive management. McDonald et al. [28] retrospectively reviewed 13 pilon fractures, of which 12 were true high-energy injuries. The technique involved application of a non-bridging three-ring circular frame, with a minimally invasive approach to articular reduction. Eleven fractures were healed by 16 weeks. There was one delayed union that required bone grafting and one non-union treated with an arthrodesis. Importantly, there were no deep infections.

Leung et al. [29] reviewed 31 distal tibial fractures with 16 cases classified as C type injuries. A protocol similar to McDonald et al. was employed with mostly non-bridging circular frames. Two patients with very comminuted C3 fractures had bridging frames to the calcaneus for 2 weeks for additional stability. All but one fracture united at an average of 13.8 weeks. One fracture was complicated by infection and required an arthrodesis. Only five patients (38 %) had good results (clinical rating system of Teeny and Wiss) possibly a reflection of the poor outcome associated with these injuries.

Vidyadhara and Rao [30] reported on 21 pilon fractures with 13 cases classified as C type injuries. Minimally invasive techniques were used for joint reduction, with limited approaches where necessary, and circular ring fixation used. The authors bridged to the calcaneus for 6 weeks in all patients with the half ring removed in the outpatient setting. All fractures united with frame removal at an average of 26.6 weeks. Seven patients developed pin-site infections which settled with local care, and one patient required pin removal at 3 months due to persistent infection. There were no deep infections.

Watson et al. [31] reviewed 107 pilon fractures treated according to a staged protocol which included initial stabilisation with calcaneal traction. Definitive treatment was based on the degree of soft tissue compromise. Forty-one patients with Tscherne grade 0 and I injuries underwent open reduction and internal fixation, with minimal incisions and low-profile implants, with most cases managed within 5 days of presentation. Sixty-four patients with Tscherne grade II and III injuries, and all open fractures underwent limited internal fixation of the articular fragments through small incisions and fine wire external fixation as definitive management. For the type C fractures in both groups, there was a significantly higher rate of complications including non-union, malunion and wound complications. They recommended small wire circular fixators for the subgroup of type C fractures. Some would argue that internal fixation when performed within 5 days of injury might have accounted for the higher complication rate, but this group was selected on the basis of the less severe soft tissue injuries.

Wang et al. [32] performed a meta-analysis of complications associated with ORIF versus limited internal fixation combined with external fixation. They included nine studies with 498 fractures. The meta-analysis found no significant differences in bone healing complications, non-union, malunion or delayed union, superficial and deep infections, arthritis symptoms or chronic osteomyelitis between the two groups.

 These studies offer some perspective when dealing with type C fracture patterns and demonstrate the low incidence of serious complications, offering some support to the use of circular ring fixation as a definitive management for these injuries.

## Management of bone defects

Segmental bone defects associated with pilon fractures have been treated with different methods. This includes bone grafting, either acutely or staged (Masquelet), vascularised fibular grafts, bone transport and acute shortening followed by lengthening.

### Bone grafting

Autologous bone grafting is used commonly for smaller bone defects and is limited primarily by the amount that can be harvested from the donor site. Allograft has been used in certain conditions in conjunction with bone morphogenetic protein (BMP); this has been demonstrated to be of value in cases of non-union with bone defects by Johnson et al. [33].

The two-staged technique described by Masquelet et al. [34] has gained popularity. During the first stage, stabilisation is performed following the bone resection and a cement spacer is inserted followed by soft tissue repair. An osteoinductive membrane is formed around the spacer. The second stage is performed a few weeks later with removal of the spacer, bone decortication and use of cancellous bone graft packing the cavity within the induced membrane. Reports for its use have been encouraging but for a mixed group of conditions; evidence for use in pilon fractures is as yet lacking.

### Vascularised fibular grafts

Use of a vascularised segment of fibula to reconstruct segmental defects of the tibia has advantages of shorter time to consolidation, increased potential for remodelling, greater resistance to infection and better long-term mechanical properties [35]. It is technically challenging and was found to have some problems including unreliable hypertrophy of the graft and late fractures [36].

### Bone shortening and staged reconstruction

Closing a metaphyseal defect by shortening the tibia is a useful option for contaminated fractures (after debridement), those associated with soft tissue loss (to facilitate closure), or when there are small segmental defects. The circular frame is applied across but not inside the zone of injury. The circular frame can then be used to lengthen the bone from a separate osteotomy and achieve bony union at the metaphyseal area simultaneously. Shortening the bone will also reduce the size of the soft tissue defect and may avoid the need for a free flap [37]. The disadvantages of this technique are the ensuing limb length discrepancy (unless lengthening is contemplated), a risk of kinking vascular structures—particularly relevant in patients who have vascular injuries and those who have had soft tissue flaps for coverage—and the risk of infection from pin tracks. Nonetheless, bone shortening and subsequent lengthening are associated with a lower complication rate than bone transport techniques [38–41].

### Bone transport

The use of an external fixator for bone transport to bridge a defect is an alternative to the shortening and staged reconstruction. It is indicated for larger defects. Circular frames are more popular than unilateral devices now for their greater stability and flexibility in the configuration. There is greater scope for correcting rotational or angular mal-alignment which may occur during the course of treatment [42]. It is usual to delay the osteotomy of bone transport by several weeks to ensure the soft tissue envelope has healed.

## Open pilon fractures

The management of open injuries follows well-established principles with urgent wound debridement and skeletal stabilisation. Uncertainty exists with the method and timing of stabilisation with some advocating early internal fixation with immediate soft tissue cover, the so-called fix-and-flap protocol. Conroy et al. [43] reported the 1-year outcome of early internal fixation (within 24 h) and soft tissue coverage in a consecutive series of 32 patients. Despite the short follow-up, encouraging radiographic and clinical outcomes were reported with a deep infection rate of 6.2 % (2 patients); both cases required amputation. Close collaboration between orthopaedic and plastic surgery services is mandatory for such a protocol to be successful, and further studies are required to confirm the wide applicability of this strategy. More commonly, these injuries are managed in two or three stages. Following skeletal stabilisation and wound debridement, soft tissue coverage is performed when deemed safe in conjunction with a plastic surgeon. Thereafter, definitive management of the fracture is performed using either internal or external fixation techniques.

Few studies report directly on the outcome of open fractures. Such injuries are often grouped together with closed fractures; when present, treatment is usually with external fixation lending to a selection bias for poor results when compared to equivalent closed injuries [25]. Gardner et al. [44] reported their results of a standard protocol used for ten open fractures with segmental bone loss. Their approach involved three stages: initial debridement and spanning external fixation; then open reduction and plate fixation with antibiotic bead placement after a delay of 1–3 weeks; and finally a planned bone grafting procedure. Nine of the ten patients in this study had healed by 24 weeks. One patient required amputation for uncontrolled infection.

## Outcomes

Successful treatment of pilon fractures is dependent on the management of the soft tissue injury, anatomical reduction in the joint surface and restoration of mechanical alignment. Whilst the relative importance of each of these factors is difficult to quantify, long-term studies generally report suboptimal outcomes in these injuries. Pollak et al. [45] studied a sample which included a large proportion of type C fractures (74 %). At 2-year follow-up, they reported lower SF-36 scores than after pelvic fractures or in patient groups with chronic illnesses such as AIDS and coronary artery disease. The outcome following external fixation was reported as the only surgeon controlled factor associated with a poor outcome; this conclusion has to be considered in light of what was termed definitive management by external fixation in this study—bridging external fixation with or without limited internal fixation—which is, today, historical and no longer a clinically relevant treatment strategy using external fixation. The protocol described in the study in fact represents the first stage of modern damage control surgery. Irrespective of the choice of fixation, the literature reports the outcome of these injuries remains suboptimal with the propensity to deteriorate over time [46–50].

## Treatment algorithm

**Fig. 6:**
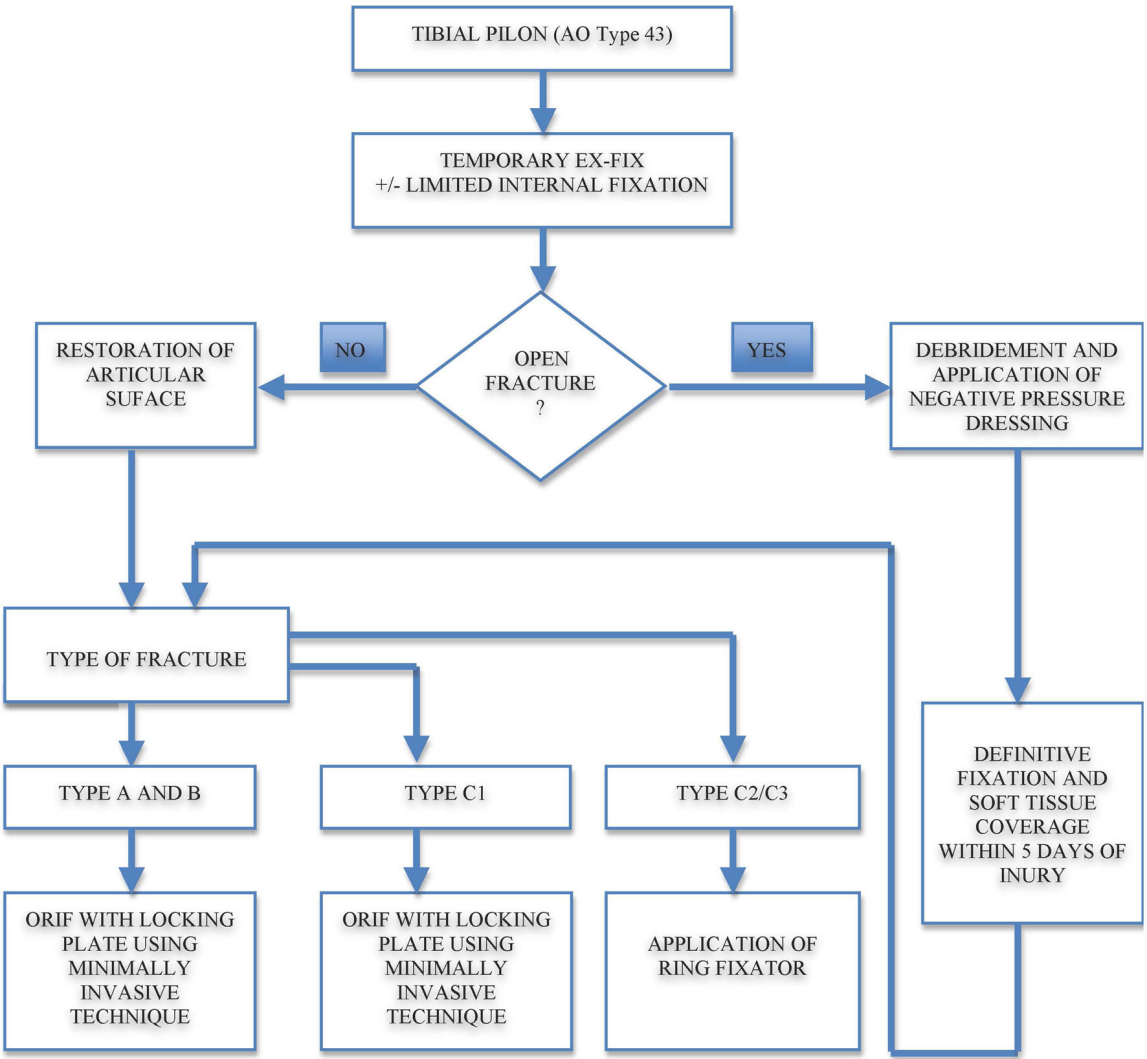
Treatment algorithm as used in the authors’ unit

Based on this review and our clinical experience, we use a treatment algorithm to guide management decisions (Fig. 6):

1. In all cases, these injuries are treated initially with a spanning external fixator.
2. With open injuries, a thorough wound debridement is performed and a spanning external fixator and temporary topical negative pressure wound dressing applied. The aim is to achieve soft tissue cover within 5 days of injury, with definitive stabilisation performed prior to soft tissue coverage or shortly after if circular fixation is used.
3. For closed injuries, we proceed to definitive management when the soft tissue swelling has settled (often 7–14 days). During this waiting period, a CT scan is performed to plan the procedure. Based on the CT, an approach is made directly onto the primary sagittal fracture line, minimising soft tissue stripping and maintaining full thickness skin flaps. The articular surface is visualised and the impacted fragments reduced under direct vision. Reconstruction of the plafond proceeds from posterior to anterior with temporary k-wires used to hold the articular reduction, and small fragment screws (cannulated and partially threaded) then applied to secure definitive stability.
4. In type C1 fractures, effectively three large articular fragments with no metaphyseal comminution, we choose to plate the tibia using minimally invasive techniques with a locking plate to bridge the articular segment to the diaphysis.
5. In type C2 or C3 fractures, an Ilizarov external fixator is used. Following articular reconstruction, a two-ring construct is applied to the tibia proximally, orthogonal to the anatomical axis. A reference wire is then passed distally, at the level of the plafond, as close as possible to the articular surface, but recognising the capsular attachments which extend 15 mm proximally. Three or four wires are inserted into the distal segment with wide crossing angles to achieve maximal stability. The distal segment is then reduced onto the two-ring proximal ring construct, with a ring-to-ring reduction in tibial alignment. The construct is extended to the hindfoot in almost all cases to provide additional stability. The hindfoot ring is removed at 6–8 weeks post-operatively in the clinic. Patients remain non-weight bearing until radiological evidence of healing is attained, and input from specialist physiotherapists prevents the development of forefoot deformities.

## Conclusion

The optimum management for tibial pilon fractures is yet undetermined. When soft tissue conditions permit and in type C1 fractures, open reduction and internal fixation with minimally invasive techniques is preferred. In type C3 fractures, a two-stage procedure of initial articular restoration and spanning external fixation followed by definitive fixation at a later stage appears to give better results [51]. However, in all these fractures, careful management of soft tissue injury holds the key to reduced complications and improved outcomes. Despite these newer surgical techniques, the long-term outcomes continue to be less than satisfactory.
